# Ameliorating standard operating procedures could reduce the risk of needle retention after acupuncture

**DOI:** 10.1097/MD.0000000000041488

**Published:** 2025-02-14

**Authors:** Hsu-Tung Hsieh, Chia-Lin Lin, Shun-Ku Lin

**Affiliations:** a Department of Chinese Medicine, Taipei City Hospital, Taipei, Taiwan, ROC; b Institute of Traditional Medicine, School of Medicine, National Yang Ming Chiao Tung University, Taipei, Taiwan, ROC; c Institute of Public Health, School of Medicine, National Yang Ming Chiao Tung University, Taipei, Taiwan, ROC; d University of Taipei, Taipei, Taiwan, ROC.

**Keywords:** acupuncture, adverse event, retained needle, standard operating procedure

## Abstract

Needle retention, where acupuncture needles are left in the patient following treatment, is a serious adverse event that can lead to infections, bleeding, and tissue damage. While rare, its potential complications render it a critical concern in acupuncture practice. Current research suggests that inadequate procedural safeguards contribute to the incidence of needle retention, yet there is limited investigation into the role of standard operating procedures (SOPs) in reducing such events. This study aimed to evaluate and compare the effects of 2 different SOPs on the incidence of needle retention in acupuncture treatment. We developed a new acupuncture procedure based on SOPs for acupuncture commonly used in hospitals in Taiwan. The main advancements included replacing paper confirmation checklists with online instant messaging systems, staff meetings before treatment, and repeated confirmations after treatment. We implemented 2 SOPs for inpatients in Taipei City Hospital from 2018 to 2021. The incidence of needle retention was calculated, and a logistic regression model was applied to estimate the odds ratio (95% confidence interval) for needle retention risk between the acupuncture SOPs. This study included 13,920 acupuncture treatments, of which 7653 followed the original SOP and 6267 followed the new SOP. The incidence of needle retention was 6.8 versus 2.2 per thousand acupuncture treatments for the original versus new SOPs, respectively. Compared with the original SOP, the new version showed a 0.41 adjusted odds ratio (95% confidence interval, 0.15–0.72; *P* = .032) of needle retention after adjusting for age, gender, body mass index, Barthel scale, and acupuncture treatment times. Ameliorating the SOPs could reduce the risk of needle retention after acupuncture. This study showed that a new acupuncture SOP, incorporating technology and better communication, significantly reduced needle retention incidents, enhancing patient safety and care quality.

## 1. Introduction

Retained needles after acupuncture represent an adverse effect that may lead to infection, pain, bleeding, and local tissue damage. In some cases, needles might break and chronically lodge in the bodies.^[1,2]^ Retained needles after acupuncture is a severe incident of medical negligence and may result in a physician facing legal penalties and damages. Cases of needle retention following acupuncture have been reported in Asia, Europe, and the Americas.^[3,4]^ Large-scale clinical tracking shows that needle retention following acupuncture occurs at the rate of 5 to 6 incidents per thousand acupuncture treatments and has a higher chance of occurrence in hospitalized patients.^[5]^ A 6-year clinic survey in Japan found that there are 0.4 failing to remove needles per thousand treatments.^[6]^ A study in the United Kingdom. The National Patient Safety Agency reports found that retained needles accounted for 31% of all adverse events.^[2]^ Past studies state that the possible risk factors of needle retention after acupuncture include physician neglect, failure of nurses to comply with standard procedures, acupuncture needles in hidden parts of the body, etc.^[4]^

The risk of needle retention during acupuncture treatments is influenced by both the underlying condition being treated and the specific acupuncture points selected. Certain points, particularly those located near vital organs, nerves, or blood vessels, pose a higher risk if not accurately targeted. For instance, needling at Jingming (BL-1), situated near the ophthalmic and angular arteries and veins, requires precise technique to avoid complications.^[7,8]^

Moreover, the type of disease can affect the likelihood of adverse events. Patients with conditions necessitating the use of “dangerous” acupuncture points may have an elevated risk of needle retention. A systematic review of acupuncture-related adverse events indicated that different acupuncture techniques could lead to varying adverse outcomes, underscoring the importance of tailored approaches based on the patient’s condition.^[7]^ Understanding these nuances is crucial for developing effective standard operating procedures (SOPs) aimed at minimizing the risk of needle retention across diverse patient populations.^[9]^

However, insufficient studies investigate the association between acupuncture SOPs and needle retention after acupuncture. Therefore, this study aimed to develop new SOPs to reduce acupuncture needle retention and evaluate the original version versus the new plate SOPs for the incidence and relative risk of needle retention following acupuncture.

## 2. Methods

A cohort study was conducted to compare 2 acupuncture SOPs regarding needle retention. The research was conducted at Taipei City Hospital, a public hospital with 7 branches, from June 1, 2018, to December 31, 2021. We collected data from the Hospital Information System and Adverse Event Notification System of Taipei City Hospital.

### 2.1. Study population

Participants in this study were hospitalized for cerebrovascular diseases (International Classification of Diseases, Ninth Revision, Clinical Modification: 430–438) or cancer (International Classification of Diseases, Ninth Revision, Clinical Modification: 140–239) in Taipei City Hospital. A neurologist or oncologist would confirm the diagnosis and provide routine modern medical treatment for cancer and cerebrovascular disease. The traditional Chinese medicine (TCM) physician explained the treatment process and risks to the patient, and the patient completed the informed consent and signed the consent form. Patients with cerebrovascular diseases receive acupuncture to increase joint mobility and reduce muscle tone. Besides, patients with cancer receive acupuncture to relieve pain and reduce the side effects of chemotherapy, such as nausea and vomiting. Because patients with cancer and stroke have poor self-care capabilities and require more complicated acupuncture treatment, these patients are a high-risk group for needle retention. Patients who refused to receive acupuncture or those with contraindications to acupuncture, such as coagulation abnormalities, skin ulcers, delirium, or agitation, were excluded from the study. The Research Ethics Committee of the Taipei City Hospital approved the study, and the relevant case number is TCHIRB-10701103-E.

### 2.2. SOPs of acupuncture

**Figure 1.**
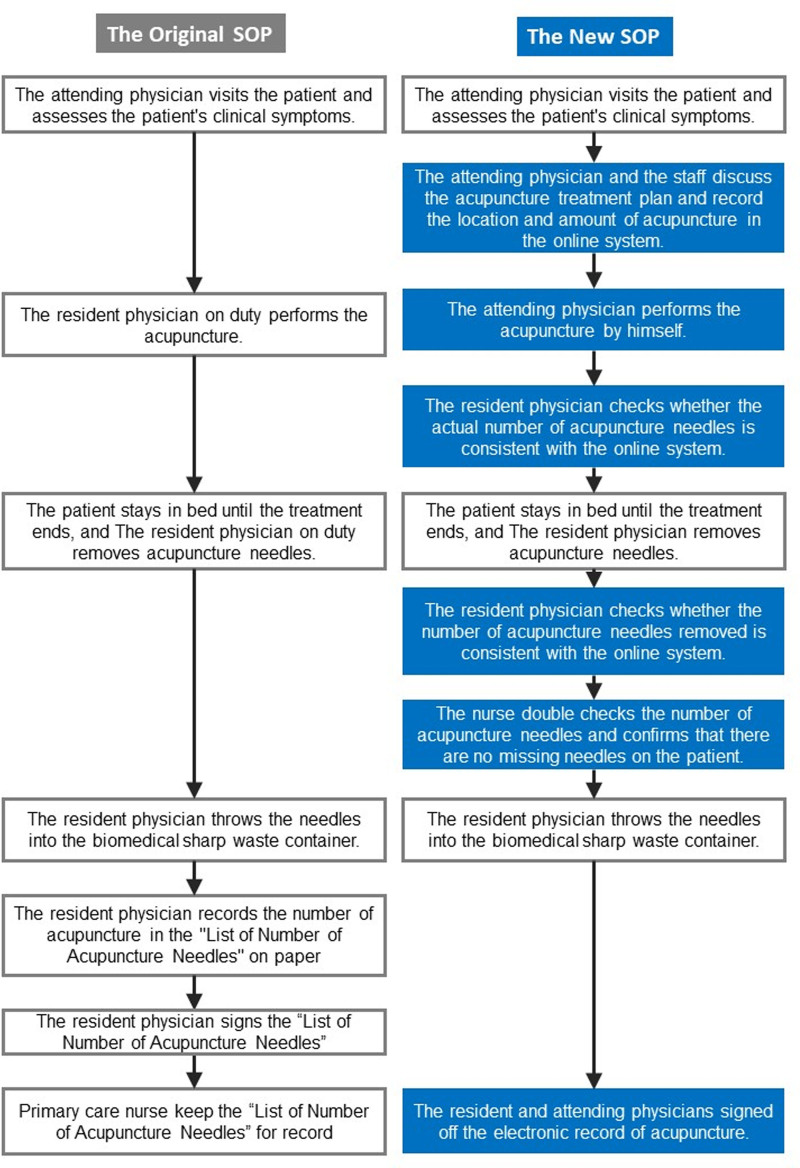
Flowcharts of the original and new standard operating procedures (SOPs) for acupuncture. The blue boxes indicate the steps added to the new version of the SOP, which includes the following: the attending physician performs each acupuncture treatment in person rather than the on-duty resident physician; the resident physician checks whether the actual number of acupuncture needles is consistent with the online system immediately after acupuncture treatment; the resident physician calculates the number of acupuncture needles removed from the patients and confirmers the number to the online system; and finally, the nurse double-checks the number of acupunctures needles and confirms that there are no missing needles.

All the attending physicians were acupuncture specialists with >5 years of clinical experience, and they followed similar acupuncture treatment guidelines to choose acupuncture points. The TCM attending physicians could determine whether to use the original SOP issued by the Taiwan Ministry of Health and Welfare or the new SOP. However, the attending physician could not replace the treatment SOP during the study period. All physicians and nurses involved in acupuncture treatment must take an 8-hour course to ensure that they can perform SOPs correctly. We listed the comparison of the 2 SOPs in Figure 1.

The original SOP follows the “Reference Guidelines for Safe Practice of Acupuncture and Moxibustion of Traditional Chinese Medicine Medical Institutions” issued by the Taiwan Ministry of Health and Welfare in 2004.^[10]^ Hospitals widely adopt the original SOP in Taiwan as the standard treatment procedure for outpatients and inpatients. In addition, the National Union of Chinese Medical Doctors’ Association required all new TCM physicians to take a 4-hour course to familiarize the original SOP before practicing acupuncture.^[11]^

The detailed steps of the original SOP are given as follows.

1. The procedure began with a hospitalized patient asking for a TCM consultation and an assigned attending physician visiting the patient and evaluating the patient’s condition.
2. The resident physician on duty performs the acupuncture. The patient stayed in bed throughout the treatment.
3. The patient stayed in bed until the treatment ended, and a resident physician removed acupuncture needles and discarded them in the biomedical sharp waste containers.
4. The resident physician records the number of acupuncture in the “List of Number of Acupuncture Needles” and signs off the list on paper.
5. Primary care nurses keep the “List of Number of Acupuncture Needles” for the record.

The Traditional Chinese Medicine Department of Taipei City Hospital developed a new SOP to reduce needle retention after acupuncture as follows.

1. The attending physician convened all medical staff for a meeting before each acupuncture session. The attending physician would explain the acupuncture treatment plan to ensure that all members understand the location and amount of acupuncture.
2. The attending physician would input the acupuncture treatment plan into the online system before the acupuncture treatment, including the acupuncture points, quantity, and duration.
3. The attending physician performed each acupuncture treatment personally, rather than the on-duty resident physician, to improve the quality and safety of the treatment.
4. The resident physician checks whether the actual number of acupuncture needles is consistent with the online system immediately after acupuncture treatment.
5. The resident physician calculates the number of acupuncture needles removed from the patients and verifies this count in the online system.
6. The nurse double-checks the number of acupuncture needles and confirms that there are no missing needles on the patient.
7. The resident and attending physicians signed off the electronic record of acupuncture.

### 2.3. Study outcomes

We followed patients from their first acupuncture treatment until they were discharged from the hospital. If patients were transferred to other wards or readmitted to the hospital, the patients would be continuously treated by the same TCM attending physician and adhere to the same SOPs. All medical staff, including physicians, nurses, functional therapists, and ward assistants, were obligated to report incidents of needle retention and notify through the adverse event reporting system. The Hospital Information System maintained records containing detailed information about retained needles, including their location, time, severity, complication, and possible reasons. We also obtained information about acupuncture from the Hospital Information System, including location, frequency, and duration. The medical information on patients, including their body mass index (BMI), length of stay, Barthel scale, and diagnosis, was also collected from the hospital information system and confirmed using the National Health Insurance Pharma-Cloud System. Two physicians independently examined all data and excluded missing and inconsistent data.

### 2.4. Statistical analysis

We compared age, BMI, acupuncture counts, hospital days, and Barthel scale for patients who received therapy using different acupuncture SOPs. We calculated the mean and standard deviation of the above variables and assessed the significance of the difference with an independent 2-sample *t* test. We performed the χ^2^ test to elevate the gender and diagnosis of difference between the 2 groups. We used the logistic regression model to estimate the needle retention risk of acupuncture. We used the odds ratio with a 95% confidence interval (CI) to represent the odds ratio between the 2 different acupuncture SOPs. To correct possible confounders, we designed 3 different statistical models: model 1 adjusted personal data such as age, gender, BMI, and Barthel scale; in model 2, we added acupuncture treatment courses to modify risk; and in model 3, we used the Cox proportional hazards model and the length of stay served as the basis for calculating the hazard ratio. We used the Statistical Analysis Software, version 9.4, developed by the SAS Institute Inc, Cary, NC, for mathematical calculations and data processing.

## 3. Results

**Figure 2.**
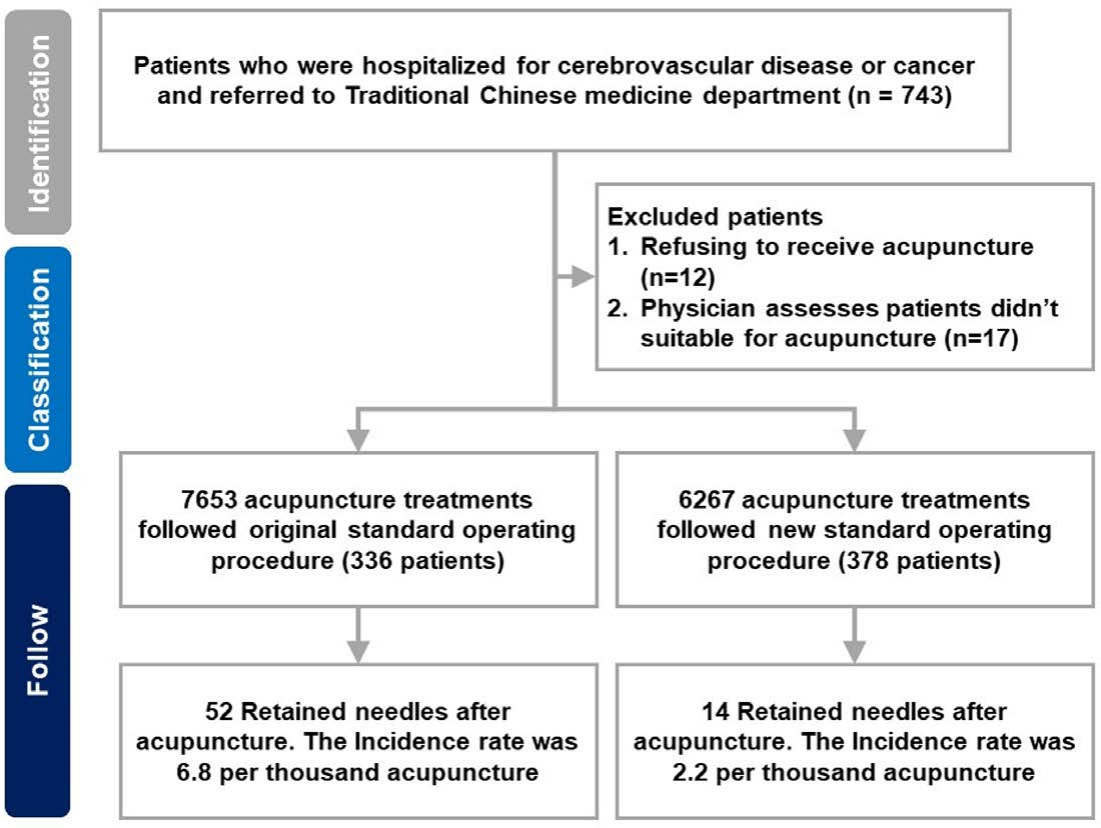
Recruitment flowchart of inpatients who received acupuncture with the different standard operating procedures (SOPs). This study included 714 patients with 13,920 acupuncture treatments, of which 7653 followed the original SOP, and 6267 followed the new SOP. The incidence of needle retention was 6.8 versus 2.2 per thousand acupuncture treatments for the original versus new SOPs, respectively.

Seven hundred forty-three patients who experienced cerebrovascular accidents or cancer were hospitalized in Taipei City Hospital and were referred to the department of TCM. Twelve patients who refused to receive acupuncture therapy and 17 who were unsuitable for acupuncture therapy withdrew from the study. Of the 714 patients who received acupuncture therapy, 336 (47.1%) patients with 7653 acupuncture treatments and 378 (52.9%) patients with 6267 acupuncture treatments followed original and new SOPs. During the study follow-up period, patients receiving the original and new SOPs experienced 52 and 14 needle retentions after acupuncture, respectively. The recruitment flowchart of patients who received acupuncture therapy with different SOPs is shown in Figure 2.

### 3.1. Baseline characteristics

Table 1 shows the basic features of patients receiving different acupuncture SOPs. Mean and standard deviation were used to represent continuous variables, including age, BMI, acupuncture counts, hospital days, and Barthel scale for both groups. The average age of the 2 groups of patients was >65 years. In addition, the Barthel scale was 57.1 ± 1.9 and 55.5 ± 1.6 for original and new SOPs, respectively. The Barthel scale <60 indicated that both groups of patients needed to rely on their caregivers to assist with daily life. Women and cerebrovascular accident patients were more than half in both groups. There was no significant difference in age, BMI, acupuncture counts, hospital days, and Barthel scale between patients who received the original and new SOPs.

**Table 1:** Basic features of patients who received different SOPs of acupuncture.

Variables	Original SOP, mean ± standard deviation	New SOP, mean ± standard deviation	*P* value of 2-sample *t* test
Age, yr	68.3 ± 8.1	69.1 ± 8.4	.42
Body mass index	23.4 ± 2.7	22.5 ± 2.8	.38
Acupuncture times	23.0 ± 7.5	21.6 ± 7.7	.19
Hospital days	69.1 ± 7.9	68.4 ± 7.8	.44
Barthel scale	57.1 ± 1.9	55.5 ± 1.6	.27
	Original SOP, n (%)	New SOP, n (%)	*P* value of χ^2^ test
Total	226 (100.0%)	151 (100.0%)	
Gender			.48
Female	196 (58.3%)	187 (55.6%)	
Male	140 (41. 7%)	149 (44.4%)	
Diagnosis			.80
Cerebrovascular accident	229 (68.1%)	232 (69.1%)	
Cancer	107 (31.9%)	104 (30.9%)	

SOP = standard operating procedure.

### 3.2. The incidence rate and risk of needle retention after acupuncture

The incidence of needle retention after acupuncture was 6.8 versus 2.2 per thousand acupuncture treatments for the original versus new SOPs. The incidence ratio of needle retention was 3.1 when comparing the original SOP with the new SOP. Needle retention per 1000 hospital days was 2.2 for the original SOP and 0.5 for the new SOP, with an incidence ratio of 4.1.

Table 2 shows the risk ratio of needle retention of the new SOP compared with the original SOP. The crude odds ratio was 0.36 (95% CI, 0.17–0.66; *P* = .01) when calculated using a logistic regression model. The adjusted odds ratio was 0.34 (95% CI, 0.18–0.63; *P* = .01) when factors such as age, gender, BMI, and Barthel scale were considered. Compared with the original SOP, the new SOP showed a 0.41 odds ratio (95% CI, 0.15–0.72; *P* = .02) of retained needles after adjusting for age, gender, BMI, Barthel scale, and the number of acupuncture treatments. We used the Cox proportional hazard model to calculate the relationship between the risk of retained needles and observed time, and the adjusted hazard ratio was 0.34 (95% CI, 0.11–0.65; *P* = .02).

**Table 2:** The risk ratio of needle retention new SOP compared with that of the original SOP.

Statistical methods	Outcome	95% confidence interval	*P* value
Crude: logistic regression model for retained needles risk after acupuncture	Crude odds ratio: 0.36	0.17–0.66	.01
Model 1: logistic regression model adjusted by age, gender, body mass index, and Barthel scale	Adjusted odds ratio: 0.34	0.18–0.63	.01
Model 2: logistic regression model adjusted by age, gender, body mass index, Barthel scale, and acupuncture treatment courses	Adjusted odds ratio: 0.41	0.15–0.72	.02
Model 3: Cox proportional hazard model adjusted by age, gender, body mass index, Barthel scale, and observed time	Adjusted hazard ratio: 0.34	0.11–0.65	.02

SOP = standard operating procedure.

## 4. Discussion

We discovered that changing the SOP of acupuncture treatments could effectively reduce the occurrence rate of retained needles after acupuncture (6.8 vs 2.2) per thousand acupuncture treatments. By replacing paper confirmation checklists with online instant messaging systems, staff meetings before treatment, and repeated confirmations after treatment, the risk of needle retention could be lowered significantly (odds ratio, 0.41 [95% CI, 0.15–0.72]; *P* = .02). Our study validates that communication between medical practitioners could be the key to avoiding needle retention after acupuncture. Implementing steps such as pretreatment communication and repeated confirmations during the treatment process can effectively reduce the risk of needle retention.

Needle retention or missing needles are also critical adverse events in surgery.^[12]^ Therefore, TCM physicians could learn from surgical experience to modify the SOP to reduce needle retention.^[13]^ For example, a TCM physician planned the number of acupuncture needles before treatment and compared it with the number of needles removed after treatment. Repeat counts by independent medical personnel also help reduce the risk of missing needles. Therefore, we invited the nurse to confirm the amount of acupuncture with the physician after the acupuncture treatment. For acupuncture needles that may remain in the body, TCM physicians could use X-rays to locate them.^[14]^

Needle retention during acupuncture often arises due to systemic procedural errors rather than the actions of individual physicians. Therefore, an accurate inventory of all needles should be conducted at the following time points during acupuncture: before acupuncture treatment (initial count), after completing acupuncture treatment, and after removing all needles. Meanwhile, all physicians should be familiar with the patient’s treatment plan to effectively increase the stability of treatment and further prevent the risk of retained needles.

Counting the number of needles during acupuncture treatment can be difficult, especially when there are many patients or when physicians are inexperienced. The complex treatment environment of acupuncture also increases the opportunity for artificial errors.^[15]^ Even with an acupuncture SOP that records needle count and a proper confirmation process, medical teams that lack communication could still cause these procedures to fail. Therefore, it is critical to confirm treatment strategy and needle count before treatment so that the entire team is privy to all information. Many measures to prevent retained needles after surgery could also be applied to reduce retained needles after acupuncture, including postsurgery X-ray inspections, radio frequency identification, microchips, and handheld scanning equipment.^[16,17]^

This study’s findings highlight the efficacy of a revised SOP in reducing needle retention during acupuncture treatments in hospitalized patients. However, the generalizability of these results warrants careful consideration. The study was conducted in a single institution specializing in TCM with highly trained practitioners, which may not reflect the practices or resources available in smaller clinics or general hospitals. Furthermore, the patient population is comprised primarily of individuals with cerebrovascular diseases and cancer, who may require more complex care and present unique risks for needle retention. While the new SOP demonstrated a significant reduction in adverse events, its applicability to outpatient settings, where procedural oversight and staff resources may differ, remains uncertain. Future research should explore multicenter trials across diverse clinical environments to evaluate the adaptability and effectiveness of these protocols.

### 4.1. Limitations

The following restrictions limit our study. Our study was limited to hospitalized patients at a single hospital. Therefore, study results cannot be extrapolated to patients in outpatient units in other hospitals or clinics. Moreover, we could not measure the physicians’ experience and the impact of the patient’s disease on retained needles. However, only well-trained and experienced TCM physicians and patients who experience cancer and stroke were approached for this study. This helped us to prevent a physician’s personal experience and skill or the patient’s condition from impacting the incidence of retained needles after acupuncture. The Hawthorne effect may also have interfered with the research, in which the individuals modify their behavior in response to the awareness of being observed.^[18]^ However, the Hawthorne effect decreases as the observation period increases. The period of this study is >3 years, which could partially reduce the risk underestimation caused by the Hawthorne effect.

Needle retention is a preventable side effect of acupuncture treatment. As responsible TCM personnel, we must maintain vigilance against retained needles following acupuncture. We encourage using new technology and working closely with medical teams to improve the current process of counting needles and treatment SOPs. In addition, a multicenter prospective study to evaluate the suitability of new technology should be performed. Finally, we must learn to work together and construct a system that could prevent the incidence of needle retention.

## 5. Conclusion

In conclusion, our study provided compelling evidence that modifying the SOPs for acupuncture treatments, by incorporating technology, improved communication, and systematic confirmations, substantially reduced the incidence of needle retention, a critical adverse event in acupuncture. The new SOP demonstrated a 59% reduction in the odds of needle retention, significantly enhancing patient safety and care quality. This suggested that enhancing procedural safeguards through technological integration and effective team coordination was essential in minimizing acupuncture-related complications. Our findings not only reinforced the importance of well-structured SOPs but also suggested that the adoption of such protocols could be instrumental in advancing the safety of acupuncture practices in clinical environments. We recommended that similar protocols be implemented in other hospitals and clinics, and further multicenter studies were necessary to validate these findings and explore additional technological advancements that could further reduce adverse events in acupuncture. In addition, exploring the role of other variables such as physician experience and patient-specific factors could have provided valuable insights into further refining acupuncture treatment safety.

## Acknowledgments

The authors thank the Department of Traditional Chinese Medicine of Taipei City Hospital for assistance with this study. In addition, they thank Shu-Yi Lin of the Department of Education and Research for assistance with statistical methods.

## Author contributions

**Conceptualization:** Hsu-Tung Hsieh, Chia-Lin Lin, Shun-Ku Lin

**Data curation:** Hsu-Tung Hsieh

**Writing - review & editing:** Hsu-Tung Hsieh, Shun-Ku Lin

**Writing - original draft:** Chia-Lin Lin

**Funding acquisition:** Shun-Ku Lin
